# The scientific basis of rational prescribing. A guide to precision clinical pharmacology based on the WHO 6-step method

**DOI:** 10.1007/s00228-020-03044-2

**Published:** 2020-11-18

**Authors:** G. A. Rongen, P. Marquet, J. M. A. van Gerven, On behalf of the EACPT research working group

**Affiliations:** 1 Department of Pharmacology and Toxicology and Department of Internal Medicine Radboudumc, PO Box 9101, 6500 HB Nijmegen, The Netherlands; 2 Centre de Biologie et de Recherche en Santé, Faculté de Médecine, Université de Limoges, 2 rue du Prof. Descottes, 87000 Limoges CEDEX, France; 3 Centrale Commissie Mensgebonden Onderzoek, PO Box 16302, 2500 BH The Hague, The Netherlands

**Keywords:** Precision clinical pharmacology, Research agenda, Eurpean Association for Clinical Pharmacology and Therapeutics (EACPT)

## Abstract

**Background and methods:**

This opinion paper expanded on the WHO “six-step approach to optimal pharmacotherapy,” by detailed exploration of the underlying pharmacological and pathophysiological principles. This exercise led to the identification of a large number of domains of research that should be addressed to make clinical pharmacology progress toward “precision clinical pharmacology,” as a prerequisite for precision medicine.

**Result:**

In order to improve clinical efficacy and safety in patient groups (to guide drug development) as well as in individuals (to guide therapeutic options and optimize clinical outcome), developments in clinical pharmacology should at least tackle the following: (1) molecular diagnostic assays to guide drug design and development and allow physicians to identify the optimal targets for therapy in the individual patient in a quick and precise manner (to guide selection of the right drug for the right patient); (2) the setting up and validation of biomarkers of target engagement and modification as predictors of clinical efficacy and safety; (3) integration of physiological PK/PD models and intermediate markers of pharmacological effects with the natural evolution of the disease to predict the drug dose that most effectively improves clinical outcome in patient groups and individuals, making use of advanced modeling technologies (building on deterministic models, machine-learning, and deep learning algorithms); (4) methodology to validate human or humanized in vitro, ex vivo, and in vivo models for their ability to predict clinical outcome with investigational therapies, including nucleic acids or recombinant genes together with vectors (including viruses or nanoparticles), cell therapy, or therapeutic vaccines; (5) methodological complements to the gold-standard, large Phase 3 randomized clinical trial to provide clinically relevant and reliable data on the efficacy and safety of all treatment options at the population level (pragmatic clinical trials), as well as in small groups of patients (as low as n = 1); (6) regulatory science, so as to optimize the ethical review process, documentation, and monitoring of clinical trials, improve efficiency, and reduce costs of clinical drug development; (7) interventions to effectively improve patient compliance and to rationalize polypharmacy for the reduction of adverse effects and the enhancement of therapeutic interactions; and (8) appraisal of the ecological and societal impact of drug use to safeguard against environmental hazards (following the “One Health” concept) and to reduce drug resistance.

**Discussion and conclusion:**

As can be seen, precision clinical pharmacology aims at being highly translational, which will require very large panels of complementary skills. Interdisciplinary collaborations, including non-clinical pharmacologists, will be key to achieve such an ambitious program.

## Introduction

Clinical pharmacology has developed from being an academic discipline of human—and veterinary—drug research and development to a broad clinical discipline that includes promoting rational pharmacotherapy [1]. Clinical pharmacologists have made major contributions to teach rational pharmacotherapy in medical curricula and to support individualized therapy by consultation and systematic medication reviews. However, they are facing increasing competition from other medical specialties and preclinical disciplines that also investigate the efficacy and safety of drugs in humans or perform research with drugs. This challenges the scientific basis and visibility of clinical pharmacology, threatens its very existence, and calls for a reflection on the unique expertise of clinical pharmacologists in, and their many contributions to, human drug research.

Clinical pharmacology has to provide a scientific basis for the regular tasks of clinical pharmacologists (clinical drug development and rational drug prescribing) and must also trigger innovations to improve their performance in daily practice. In clinical practice, the WHO-enforced six-step method has proven to be a valuable tool to systematically choose the best pharmacotherapy available and the most appropriate dose for each individual patient. We argue that the scientific principles that underlie the WHO six-step method align closely with clinical practice and are therefore highly clinically relevant. Therefore, we decided to use the six-step method to make a detailed exploration of the underlying pharmacological and pathophysiological principles and an inventory of the scientific questions that arise from current clinical pharmacological practice. This perspective can be used as a guide to approach the holy grail of clinical pharmacology: 100% efficacy of pharmacotherapy with 0% adverse effects, for all treated patients. We have coined the term “precision clinical pharmacology” for this systematic approach and the science and technology that support this process, as a prerequisite for “precision medicine” that covers a broader scope of diagnostic and therapeutic modalities, which does not specifically involve the important contributions of (clinical) pharmacological processes in optimized drug therapy [2].

## Step 1: what is the patient’s problem?

Rational therapy starts with defining the exact problem that causes the complaints of the patient. Precision clinical pharmacology expands this to an analysis of the underlying molecular biological and pathophysiological derangements. Modern biomedical science allows us to identify more often and with increased precision the molecular pathways that cause diseases in individual patients. Ideally, these insights will give rise to the design of drugs with specific mechanisms of action. Improved pathobiological understanding also results in more precise diagnosis and therapy that targets the biochemical mechanisms involved in the pathological condition of a given patient. For example, 25 years ago, we diagnosed lung cancer either as small cell lung cancer or non-small cell lung cancer. This simple classification has evolved to a much more precise diagnosis at the level of the mutated protein that is precisely involved, with consequences for optimal therapy (“targeted therapy”) [3–5]. This development implies that clinical pharmacologists should at least know the molecular pathways possibly involved in the pathophysiology of a disease and should be able to apply the outcomes of these molecular diagnostic tests to treat the disease with optimal efficacy and safety. Scientists in clinical and non-clinical pharmacology should also contribute to the discovery of new pathways involved in diseases and identify new targets for individualized therapy. Research in this field should also aim at developing and validating diagnostic tests to pinpoint these molecular pathways in individual patients. An interesting example that provides a glimpse of the future is the molecular characterization and drug-sensitivity testing of circulating cancer cells (“liquid biopsy”) [6].

## Step 2: what is the aim of the therapy?

In traditional clinical pharmacology, curation, palliation, and prevention are considered the main possible aims of (pharmaco)therapy. In precision clinical pharmacology, the aim of therapy is defined in molecular and pathophysiological terms by measuring directly in patients, target engagement, and activity modulation by the therapy. The assumption is that these intermediate markers of efficacy will allow optimizing the drug effects and predict the impact on patient outcomes. In many therapeutic areas, this approach has increasingly replaced symptom-based therapy, particularly for disease-modifying or (secondary) preventive treatments. Classical examples are blood pressure, serum glucose, and plasma cholesterol/lipoproteins monitoring to guide therapies aimed at reducing the risk of acute cardiovascular events such as myocardial infarction or stroke. However, the more we learn about the pathophysiology of the diseases and improve our skills to identify the exact pathophysiological pathways involved in the individual patient, the more precisely we will be able to describe the aim of therapy. For example, when we target LDL-cholesterol to prevent atherosclerosis in a patient by prescribing a PCSK9 inhibitor, we may not only aim to reduce LDL-cholesterol but also to reduce plasma (free) PCSK9 concentration [7] and use this marker to monitor target engagement and understand whether increasing the dose of the PCSK9 inhibitor in case of insufficient LDL-cholesterol reduction is rational (see also step 6). More advanced technologies include imaging techniques (for example PET scans to detect target (immune) cells in tumors [8]) or protein/gene expression in biological samples such as tumor biopsies, blood, cerebrospinal fluid, or urine, in response to therapy. Clinical pharmacologists should play a critical role in finding and validating (by means of specific clinical trials) these intermediate outcome measures of pharmacological activity, which should subsequently guide modifications in drug dose or type of intervention and improve clinical outcome. This will assure that the aims of therapy defined in molecular or cellular terms are still aligned with clinical outcome.

Apart from these technological improvements, tools should also be developed—or improved—to explore the patient’s aims and expectations about possible pharmacological interventions, as should the caregivers’ skills to communicate realistic outcomes of the disease and scenarios with or without intervention. Real-life monitoring (using apps or other wearable or implantable technologies) will provide important information on fluctuations of treatment effects in relation to drug administration. This will allow improving shared decision making and dose adaptation with the ultimate goal of improving patient satisfaction and compliance. Research to develop, improve, and validate tools to optimize shared decision making should also be within the research scope of clinical pharmacology and could be considered as part of “step 2.”

## Step 3: what are the therapeutic options?

Data on efficacy and safety of the therapeutic options have to be compared and shared with the patient. These data should be derived from high-quality clinical research. Classically, this research includes the well-known phases of drug investigation with the highest level of evidence derived from double-blind, randomized controlled trials with relevant patient-related outcomes that correspond with the aims defined in step 2. Randomized clinical trials offer information about the numbers needed to effectively treat (or harm) patients, which even for effective treatments usually indicate that the proportion of patients who will respond well to the therapy is smaller than the proportion of non-responders. Moreover, since generally clinical trials study one new therapy on top of standard care at a time, without comparison with the other available therapeutic options, information is often lacking to help clinicians make evidence-based choices between treatment options with roughly similar effects in trials. Furthermore, the therapeutic history and responses to previous interventions often differ between patient populations included in available trials and the patient who seeks advice, complicating the translation of trial results to the individual patient. This conundrum is and will be made worse for individualized therapies based on the precise molecular pathophysiology of the disease. This reduces the number of patients eligible for this kind of trial; hence, their statistical power to demonstrate efficacy and safety, or to compare treatments. At the same time, the number of required trials increases. This results in a paradox: the more precise our mitigation of the pathophysiology of the disease and the more efficacious these therapies for the individuals with matching disease characteristics, the more difficult (or even impossible) it will be to prove safety and efficacy for all treatment options by properly sized RCTs. These problems of evidence-based medicine offer a significant challenge for current clinical pharmacologists: to propose and validate innovative methodology of drug research so as to provide relevant and reliable data on clinical drug efficacy and safety in small groups of patients. Often, biomarkers that detect efficacy of the candidate drug to engage and influence the (molecular) target are used. The ability of these read-outs to predict relevant patient-related outcomes must be validated, preferably no later than during the development of the drug. Alternative trial designs (such as “*n*-of-1 trials” and comparison between standard therapy versus target-directed “precision” therapy) may also help understand the clinical usability of precision medicines [9]. These strategies have in common that they focus on the evaluation of efficacy and not necessarily on the detection of adverse reactions to the drug. Clinical trials yield numbers needed to harm but offer poor predictions about which patients are actually at risk. Therefore, we must also develop research strategies (including biomarkers) that predict off-target adverse reactions in individual patients. Preclinical human models of diseases using human cells or tissues in vitro, or even cultured organoids derived from human stem cells have recently been developed and have a high potential to become part of the solution for this important aspect of precision pharmacology, to reliably predict individual drug responses [10]. Even so, the clinical validity of these models must be evaluated and validated empirically as predictors of patient-related outcome.

Consequently, we have to strengthen the scientific basis of our discipline with innovative methodologies to efficiently validate biomarkers of pharmacological activity, and human translational models (in vitro, ex vivo, and in vivo) that predict the clinical outcome of investigational drugs. These methods are not meant to replace randomized clinical trials with relevant patient-related outcomes (“Phase 3 trials”). Instead, these “hard outcome” trials are an opportunity to validate these biomarkers and human translational models as predictors of outcome (benefit and harm) and for the subsequent adaptation of doses or selection of patients who will benefit most from the intervention. Furthermore, these validated methods will help selecting the most appropriate treatment for an individual patient, among the different evidence-based interventions (step 2).

Another challenge for our scientific discipline of clinical pharmacology is the rapid expansion of the modes of interventions on the pathophysiology of diseases. Traditionally, clinical pharmacologists have focused their research on molecules (small or large) that interact with target proteins (receptors, enzymes, transporters, and channels) and influence their activity. However, as molecular science, immunology, and cell biology develop, new targets are probed for intervention such as mRNA and DNA or its methylation, influencing protein expression and protein function. Not only are “simple” molecules designed to interfere with therapeutic targets, but complex structures such as nanoparticles, viruses, bacteria, and even cells are engineered as therapeutic tools or delivery systems of (small) molecules to engage and interfere with target molecules or cells. In addition to these therapies directly influencing their targets, innovative vaccines or CAR-T cells aimed at enhancing or silencing specific immune responses against a molecular or cellular target are increasingly used in the treatment against various diseases such as cancer, auto-immune diseases, infections, or neurodegenerative diseases (including dementia) [11, 12]. The scientific methodology and philosophy that clinical pharmacologists have developed for the evaluation of efficacy and safety of traditional drugs can (and should!) also be applied to the clinical development of these more advanced therapy medicinal products (ATMPs). Specifically, the complexity and dynamics of cellular therapies challenges clinical pharmacologists to develop new methods to investigate their mode of action and biological effects, and to make rational (mechanism/pharmacology-based) choices between the growing number of alternative approaches. Prediction of safety and efficacy for these therapies has taken new dimensions, including genetic modifications, cellular expansion and differentiation, tissue regeneration, permanent residence, and vertical transmission of genetically modified cells or micro-organisms, which requires new preclinical (humanized) models and validated tools to translate preclinical observations to humans in vivo. Furthermore, ATMPs also demand different approaches of the other steps of the “advanced six-step” strategy. Vaccines and therapies with (replicating) cells challenge our classical concepts of ADME, dose-response relations, and (physiological) PK/PD modeling. These mathematical approaches will need to be expanded with advanced disease models, in order to improve the design of studies with therapies that modify the course of slowly developing or recurrent conditions.

## Step 4: patient characteristics that influence therapeutic options

In addition to the disease itself and its detailed pathophysiological classification (advanced step 1), co-morbidities, co-medications, and individual characteristics of drug absorption, distribution, metabolism and elimination (ADME), age, pregnancy, and other relevant determinants should guide the choice of the pharmacotherapy (nature and dose) with the best risk-benefit balance expected. For this step, our scientific discipline should reveal the precise mechanisms of ADME in humans and how constitution (body size and weight, genetic variations), environmental factors (including the microbioma), co-medications, and co-morbidities affect ADME. In addition, attention should be paid to potential interactions between individual (patho)physiological properties (related to age, sex, cognition, organ function, genetic characteristics, etc.) and the pharmacodynamics of the proposed therapy. Predicting dose-response relationships for therapeutic and adverse effects in individual patients can be based on methods developed as part of research described in steps 2–3, but now applied to interactions with individual patient characteristics (predictors), instead of groups of patients with the same disease mechanism and limited variability in co-morbidities or demographics (descriptive models). Such methods can entail deterministic models combining disease evolution and the drug PK/PD, or empirical models derived from biostatistical (“artificial intelligence”) analyses of very large patient databases, and possibly both types combined. Their ability to predict accurately the effect of the intervention in individual patients (instead of groups) then needs to be evaluated and validated. In particular, the question arises whether patient outcome is improved when individual therapy is guided by these tools. An example of this type of research has recently been provided by RCTs that specifically addressed the hypothesis that pharmacogenetic tests prior to therapy with coumarin-derivatives or clopidogrel improved clinical outcome [13, 14]. Typically, these trials need large sample sizes to reach sufficient statistical power, facing again the main scientific challenge for our discipline: because of the many existing genetic variants and drug-gene interactions, the number of eligible patients may not be sufficient to validate all the genetic tests for their ability to predict clinical dose-effect relationships and their efficacy, when actioned, at improving clinical outcome. More generally, this applies to all the tests or strategies that are (or have been) developed to predict individual dose-responses (in terms of efficacy or adverse events), from trendy innovative techniques based on in vitro cultures of tissues/organs derived from individual patient’s pluripotent stem cells to very old-fashioned techniques such as renal function estimation, often used in clinical practice for this purpose. Therefore, it is also the clinical pharmacologists’ responsibility to fathom out the minimal validation requirements for each of these techniques, so as to provide sufficient empirical evidence to secure and foster application in clinical practice.

## Step 5: write out the prescription and give patient instruction and information

The patient and drug characteristics have already been carefully analyzed in steps 1 (disease characteristics), 2 (therapeutic objectives), 3 (mechanistic and kinetic properties of treatment options), and 4 (individual variabilities). In step 5, these factors need to be combined into an optimal individual selection of the drug and dosing schedule. Moreover, at this stage of rational prescribing, affordability (cost-effectiveness) and feasibility (compliance of the patient with the therapy chosen) should also be considered.

Reducing clinical developmental costs is an important challenge that should be addressed scientifically by clinical pharmacologists: how can we contribute to an effective drug development process with minimal attrition and optimal added therapeutic value at reasonable and affordable costs? How can we help provide high-grade clinical evidence as efficiently as possible? The answers to these questions should be sought not only in optimal trial designs (which have implicitly also been addressed in the previous steps of rational prescribing) but also in balanced application of good clinical practice (GCP). How can we tackle the hurdles that contribute to a large proportion of failed drug developments, such as insufficient target engagement, demographic or genetic variability, disease-drug or drug-drug interactions, and disease heterogeneity? These questions should be at the heart of drug development and review processes, and largely addressed by clinical trials. However, the results of these more complicated mechanistic studies may also be more difficult to interpret than “hard endpoints” in large RCTs, particularly if they result in multiple subgroups and endpoints. Moreover, a better understanding of why drugs fail for certain patients and work for others, or confirmation of post hoc findings from trials, may well complicate patient selection and increase development costs with increased drug pricing as a consequence. Empirical approaches to drug and dose optimization (e.g., guideline-based selection followed by dose titration, or drug switching based on patient responses) may be simpler and more appealing than treatment decision based on mechanistic considerations and biomarkers. The consequence of this traditional “evidence-based” approach however is a larger number needed to treat, leading to a more uncertain individual prescription. In contrast to this traditional “evidence-based approach,” implementation of precision clinical pharmacology will increase the reliability and replicability of study results [15] and make prescriptions safer and more effective in clinical practice. However, such a promise needs both a scientific basis and empirical evidence. Double-blind randomized controlled Phase 3 trials offer the best possible proof of efficacy and are most appropriate to validate precision clinical pharmacology as a predictor and enhancer of clinical outcome. However, such trials are increasingly thwarted by insufficient statistical power as a result of more precise and personalized characterization of the pathophysiology of the disease, pharmacodynamics, and pharmacokinetics (see also previous steps). Therefore, our scientific discipline will have to develop new and efficient methods to provide empirical evidence. In this regard, recent developments in information technology (introduction of electronic patient dossiers (EPD), artificial intelligence, and “big data” applications) provide opportunities to continuously monitor the outcome of our clinical care and the impact of alterations in this care (such as implementation of precision clinical pharmacology) on outcome (“integrated health care systems”) [16]. Furthermore, these systems support efficient implementation in routine clinical practice of large-scale register-based randomized comparative trials which may overcome the above-mentioned power problems in the validation of precision clinical pharmacology [17, 18].

“Cost” should be translated not only as “money spent” but also in terms of ecological impact of fabrication and use of medical products, which also affects human well-being [19] and the planet. The importance of the “One Health” principle has recently been stressed dramatically by the emergence of the COVID-19 pandemic, where human behaviors may have facilitated transmission of a highly virulent virus between animals and humans [20]. The increasing world-wide usage of ever more potent drugs enhances the risks of environmental exposure to active drugs or metabolites, which could lead to resistance or other indirect health effects. Therefore, the ecological impact of medical therapies should be included in cost-effectiveness evaluations and the ways to minimize it better investigated. To address this challenge, we will need multidisciplinary research involving human and veterinary pharmacologists and toxicologists, ecotoxicologists and One Health experts. Clinical pharmacologists will contribute essential knowledge about biodistribution, mechanisms of action, metabolite formation, and potential biological adverse effects of the compounds. The immediate relevance for prescription is by giving clear instructions on proper usage and disposal, and providing a dosing schedule that facilitates adherence and prevents waste.

With respect to compliance, we need to improve our knowledge about the factors that influence patient adherence to medical care in general and to drug intake in particular, and how we could modify this effectively. Reducing polypharmacy is an important element in improving compliance, and also patient safety. On the other hand, well-thought-out drug combinations can also improve the treatment benefit-risk balance for certain diseases, by cumulating the therapeutic effects and reducing the exposure-related adverse effects. Rational polypharmacy is a particularly difficult topic for evidenced-based drug development and pharmacotherapy because each possible combination increases the complexity of traditional RCTs. Advanced pharmacotherapeutic considerations can provide guidance to rational polypharmacy. Most of the previous “advanced” steps can also be applied to combinations of drugs. This can be achieved through careful analysis of the different mechanisms of action that can be used to interfere more efficiently with multifactorial diseases or to interrupt complex pathophysiological cascades. In the same vein, the review of additive on- or off-target effects of polypharmacy can help predict potential aggravation of adverse effects. Further development of rational polypharmacy of existing drugs, based on sound pharmacological mechanisms and principles, can make important contributions toward the reduction of the burden of adverse events and potentially improve clinical outcomes at limited costs.

## Step 6: monitoring of efficacy and safety of the therapy

This final step of rational prescribing acknowledges the fact that, despite our best efforts to improve precision in predicting efficacy and safety of our medical interventions in an individual patient, we will never attain the holy grain of healing every patient without adverse effects, and residual errors will persist. Therefore, each prescription should be considered an experiment in itself that needs close monitoring to confirm efficacy and safety at the individual scale. The challenge for clinical pharmacologists is to design methods to detect, as early as possible, deviations from predictions and their cause and to guide appropriate actions, so as to prevent unwanted clinical outcomes. For this purpose, measures of drug exposure and measures of target(s), engagement(s), and function(s) (i.e., biomarkers of efficacy and safety) that have been validated in the drug development phases as predictors of pharmacological and toxicological effects at the population level should be formally evaluated for their ability to guide therapy adjustments and improve clinical outcome in individuals. This should provide clinicians with tools to answer the important questions: why did a drug fail to perform as intended? Did the drug reach its action site? Did it have its intended pharmacological effect? Was the effect sufficient to correct the pathophysiological derangement? And if not, why not and how can this be corrected? These questions are important, not only for individual pharmacotherapy but also for improved drug development. The precision clinical pharmacology approaches described in this article are meant to improve this process, hence representing an important way forward to the applied science of clinical pharmacology.

## Conclusion

This paper built on the practical logic of the WHO six-step approach to optimal pharmacotherapy, by expanding on the underlying pharmacological and pathophysiological principles. This shows that the clinically relevant domains of research which should be addressed to provide a sound scientific basis for rational development and prescribing of medical therapeutics—the main mission of clinical pharmacology as a scientific and medical discipline and a prerequisite for “precision medicine”—are very vast, going from basic science to patient education, through regulation and environmental toxicology. Actually, “precision clinical pharmacology” has to be highly translational, and this will require very large panels of complementary skills. Inter-disciplinary collaborations including non-clinical pharmacologists will be key to achieve such an ambitious program. These collaborations can be structured around the optimization of ever more targeted therapies in individual patients with diseases that are increasingly well understood.
